# Diaphragmatic dysfunction is associated with postoperative pulmonary complications and phrenic nerve paresis in patients undergoing thoracic surgery

**DOI:** 10.1007/s00540-024-03325-5

**Published:** 2024-03-28

**Authors:** Jesper Nørskov, Søren Helbo Skaarup, Morten Bendixen, Hatice Tankisi, Amalie Lambert Mørkved, Peter Juhl-Olsen

**Affiliations:** 1 Department of Cardiothoracic- and Vascular Surgery, Aarhus University Hospital, Palle Juul-Jensens Boulevard 99, 8200 Aarhus N, Denmark; 2 Department of Respiratory Diseases and Allergy, Aarhus University Hospital, Aarhus N, Denmark; 3 Department of Clinical Neurophysiology, Aarhus University Hospital, Aarhus N, Denmark; 4 Department of Clinical Medicine, Aarhus University Hospital, Aarhus N, Denmark

**Keywords:** Diaphragmatic dysfunction, Phrenic nerve paresis, Postoperative pulmonary complications.

## Abstract

**Purpose:**

We aimed to quantify perioperative changes in diaphragmatic function and phrenic nerve conduction in patients undergoing routine thoracic surgery.

**Methods:**

A prospective observational study was performed in patients undergoing esophageal resection or pulmonary lobectomy. Examinations were carried out the day prior to surgery, 3 days and 10–14 days after surgery. Endpoints for diaphragmatic function included ultrasonographic measurements of diaphragmatic excursion and thickening fraction. Endpoints for phrenic nerve conduction included baseline-to-peak amplitude, peak-to-peak amplitude, and transmission delay. Measurements were assessed on both the surgical side and the non-surgical side of the thorax.

**Results:**

Forty patients were included in the study. Significant reductions in diaphragmatic excursion were seen on the surgical side of the thorax for all excursion measures (posterior part of the right hemidiaphragm, *p* < 0.001; hemidiaphragmatic top point, *p* < 0.001; change in intrathoracic area, *p* < 0.001). Significant changes were seen for all phrenic nerve measures (baseline-to-peak amplitude, *p* < 0.001; peak-to-peak amplitude, *p* < 0.001; transmission delay, *p* = 0.041) on the surgical side. However, significant changes were also seen on the non-surgical side for all phrenic nerve measures (baseline-to-peak amplitude, *p* < 0.001; peak-to-peak amplitude, *p* < 0.001; transmission delay, *p* = 0.022). A postoperative reduction in posterior diaphragmatic excursion of more than 50% was significantly associated with postoperative pulmonary complications (coefficient: 2.69 (95% CI [1.38, 4.01], *p* < 0.001).

**Conclusion:**

Thoracic surgery caused a significant unilateral reduction in diaphragmatic excursion on the surgical side of the thorax, which was accompanied by significant changes in phrenic nerve conduction. However, phrenic nerve conduction was also significantly affected on the non-surgical side to a lesser extent, which was not mirrored in diaphragmatic excursion. Our findings suggest that phrenic nerve paresis plays a role in postoperative diaphragmatic dysfunction, which may be a contributing factor in the pathogenesis of postoperative pulmonary complications.

**Clinical trials registration number:**

NCT04507594.

**Supplementary Information:**

The online version contains supplementary material available at 10.1007/s00540-024-03325-5.

## Introduction

Postoperative pulmonary complications (PPCs) pose a major challenge after thoracic surgery and are associated with prolonged hospitalization, increased healthcare costs, ICU-admissions, and higher mortality [1–4]. The risk of PPCs is reported to be significantly higher in patients with postoperative diaphragmatic dysfunction [5]. However, the etiologies contributing to postoperative diaphragmatic dysfunction remain unclear.

Diaphragmatic dysfunction may in part be caused by temporary or permanent paresis of the phrenic nerve after thoracic surgery [6]. While phrenic nerve paresis is well recognized after cardiac surgery [7], the extent of phrenic nerve damage and subsequently diaphragmatic dysfunction remains poorly described after classic thoracic surgery procedures.

Diaphragmatic function can easily be quantified using novel ultrasound techniques to measure the excursion and thickening fraction in different regions of the diaphragmatic dome. Additionally, phrenic nerve conduction studies can measure the summation of functional neuronal activity and transmission time within the phrenic nerve.

Our primary hypothesis was that diaphragmatic excursion as measured with posterior hemidiaphragmatic excursion was reduced after thoracic surgery. We aimed to elucidate the perioperative changes in ultrasound measures of diaphragmatic function and measures of phrenic nerve conduction after lobectomy and esophageal resection surgery. Further, we aimed to describe the associations of common ultrasound measures of diaphragmatic function with PPCs.

## Materials and methods

### Study design and patients

This prospective observational study was approved by the National Committee on Health Research Ethics (M-2019-101-22), published on ClinicalTrials.gov prior to enrollment (NCT04507594) and conducted at Department of Cardiothoracic- and Vascular Surgery, Aarhus University Hospital, Denmark.

Patients older than 18 years and scheduled for open surgical- or video-assisted esophageal resection surgery or pulmonary lobectomy were eligible for inclusion. Exclusion criteria were known preoperative diaphragmatic dysfunction including phrenic nerve paresis, any neuromuscular disease, pleural effusion > 1 cm, pneumothorax, and body mass index above 40 kg/m^2^. Participation was conditional upon written informed consent.

### Study protocol

**Fig. 1:**
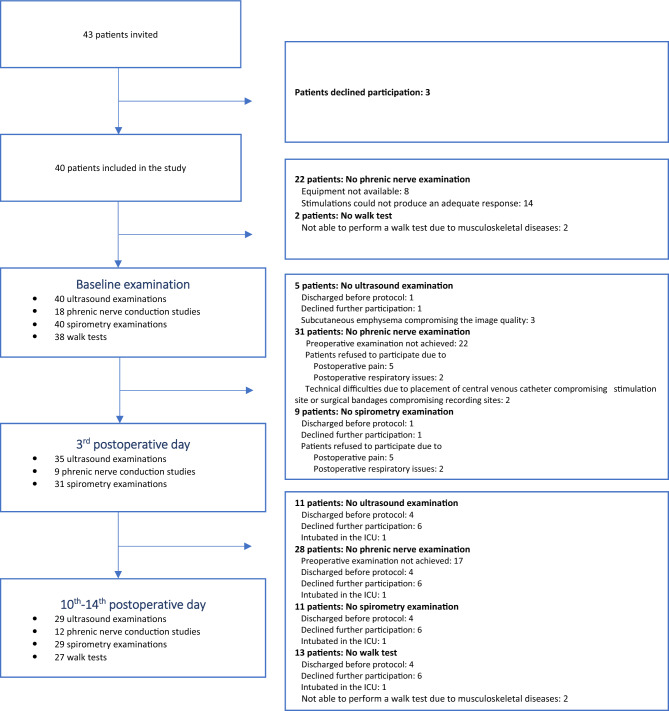
Flowchart of patient examinations during the study

All patients underwent ultrasound examinations of the diaphragm at baseline (the day prior to surgery), 3 days after surgery and 10–14 days after surgery as illustrated in Fig. 1. Phrenic nerve conduction measurements were performed at the same time points in a subset of patients. Participation was not conditional on participation in phrenic nerve conduction measurements as stimulation of the nerve may cause discomfort.

### Anesthesia

Patients were anesthetized with propofol, fentanyl, and rocuronium, and anesthesia was maintained with propofol and fentanyl. As a rule, patients scheduled for open surgery had an epidural catheter. Patients were intubated with a double-lumen tracheal tube and ventilated with tidal volumes of 6–8 mL/kg during two-lung ventilation and 5 mL/kg during one-lung ventilation. Peak inspiratory pressures were kept below 30 cmH_2_O. Epidural analgesia was routinely maintained for 4–5 days after surgery for patients undergoing esophageal resection and following clinical judgment in open lobectomy patients.

### Surgical procedures

Lobectomies were done as either Video-assisted thoracoscopic surgery (VATS) or open surgical procedures. The VATS approach was performed as a three-port technique (Copenhagen approach [8]), where anatomical dissection of vessels and bronchus, separation of structures using endoscopic stapling, and sampling of at least three N2 lymph-node stations were performed. Open surgical procedures were performed as an anterolateral thoracotomy.

Esophageal resections were performed with either a full scopic technique, hybrid- or open/open procedures. The endoscopic technique in the abdomen was performed with five ports and insufflation of CO_2_, whereas the open technique was performed as a midline laparotomy. The right-sided VATS technique was performed using five ports and insufflation of CO_2_. Open thoracic surgery was performed through a right-sided anterolateral thoracotomy. The stomach was first freed from surrounding structures, and the neo-esophagus was made from the major curvature. The neo-esophagus was pulled through the diaphragm into the thoracic cavity, and the esophagus divided with tumor margin of at least 7 cm. An end-to-side anastomosis was made between the esophagus and the neo-esophagus using a circular stapler.

### Ultrasound measurements

Ultrasound measurements were performed using a GE Vivid S70 ultrasound machine (GE Healthcare, Horten, Norway) with the patient in a semi-recumbent position. Diaphragmatic excursion measures and thickening fractions were obtained using an M5Sc-D cardiac sector transducer and a 9L linear transducer (GE Healthcare), respectively. Cine loops were stored for subsequent analyses by an expert sonographer blinded to all other study data. Diaphragmatic excursion was measured with three different ultrasound techniques to quantify excursion in different diaphragmatic regions. In addition, diaphragmatic thickening was measured as an index of muscle contraction.

#### Diaphragmatic excursion

##### Excursion of the posterior part of the right hemidiaphragm

**Fig. 2:**
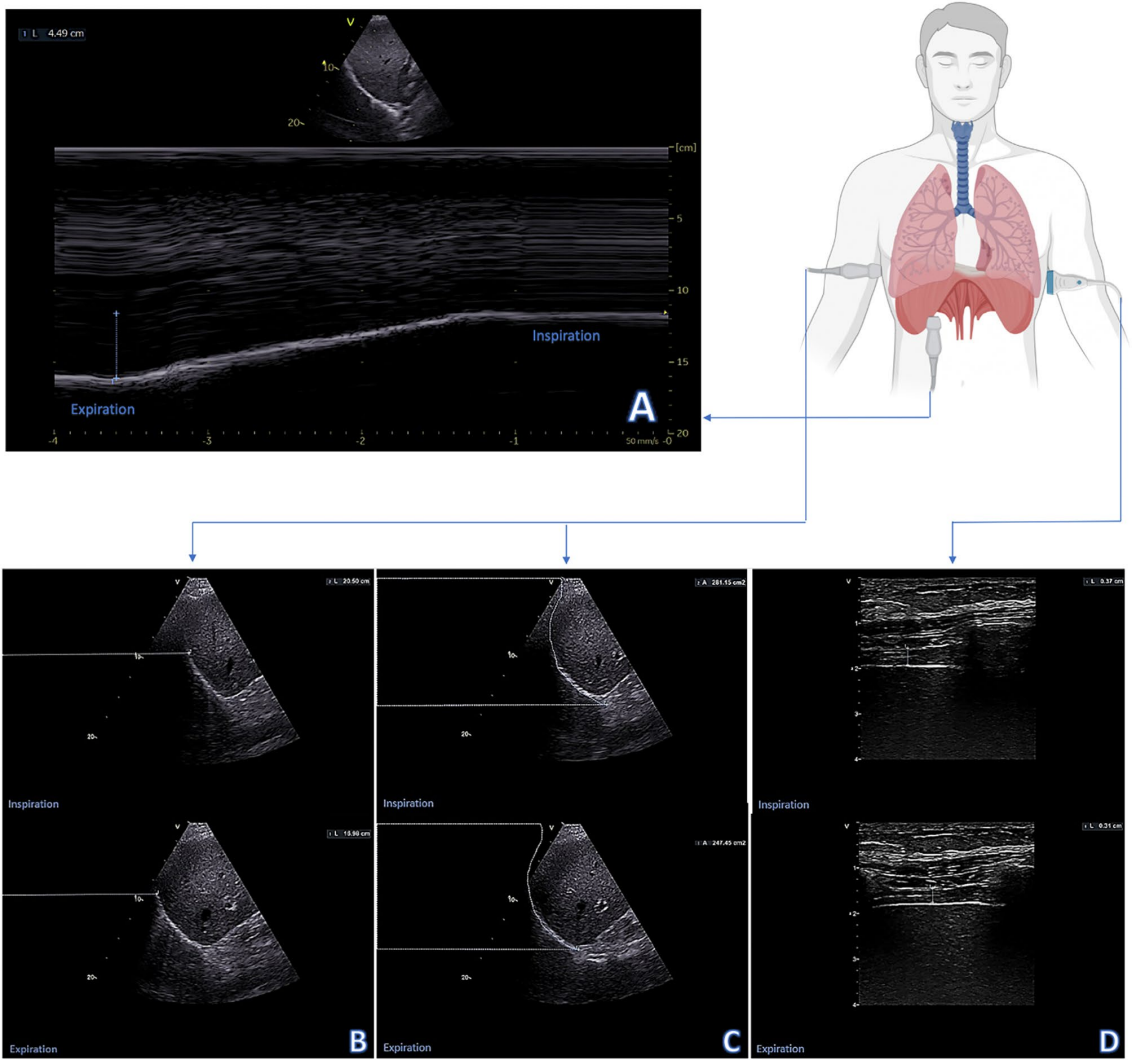
Ultrasonographic endpoint. **A** Excursion of the posterior part of the right hemidiaphragm performed between the midaxillary and midclavicular line. **B** Excursion of the hemidiaphragmatic top point in the midaxillary line. **C** Change in intrathoracic area of the hemidiaphragm performed in the midaxillary line. **D** Diaphragmatic thickness of the right hemidiaphragm

Excursion of the posterior part of the right hemidiaphragmatic dome was quantified using M-mode as described previously [9]. The transducer was placed below the right costal margin between the midclavicular and midaxillary line and tilted cranially, medially, and dorsally to achieve a perpendicular view of the posterior part of the hemidiaphragm. From this view, the hemidiaphragmatic exploration line was identified, and diaphragmatic excursion was recorded as a one-dimensional, downward-anterior movement using M-mode (Fig. 2A). The left hemidiaphragm was not assessed with this technique due to frequent artefacts caused by air in the stomach.

##### Excursion of the hemidiaphragmatic top point

For assessment of the hemidiaphragmatic top point excursion, the transducer was placed in the midaxillary line to identify the right or left hemidiaphragm, respectively [10]. The transducer was angled vertically, so the diaphragmatic movement could be followed as a cranio-caudal movement (Fig. 2B). Subsequently, B-mode cine loops were stored for The Area Method (below).

##### Change in intrathoracic area

The Area Method was performed by tracing the hemidiaphragmatic domes from inspiration to expiration, thus recording the excursion of the hemidiaphragm as a change in area [11]. Hence, diaphragmatic movement was isolated as the only change in intrathoracic area and the difference in intrathoracic area could be calculated by subtracting the area at inspiration from the area at expiration (Fig. 2C).

#### Diaphragmatic thickening fraction

Measurement of diaphragmatic thickness was performed with the transducer placed perpendicularly to the diaphragmatic muscle layers in the midaxillary line 0.5–2 cm below the costodiaphragmatic recess to observe the diaphragm’s zone of apposition [12]. The diaphragm was identified as a 3-layered structure consisting of 2 hyperechogenic parts, the diaphragmatic pleura and peritoneum, with the hypoechogenic muscle layer in between. We assessed the diaphragmatic thickness from the inner layer of the diaphragmatic pleura to the inner layer of the diaphragmatic peritoneum at the end of inspiration (DT_EI_,) and the end of expiration (DT_EE_), and calculated the diaphragmatic thickening fraction (DTf) as: ${\text{DTf}}=\frac{{{\text{DT}}}_{{\text{EI}}}-{{\text{DT}}}_{{\text{EE}}}}{{{\text{DT}}}_{{\text{EE}}}}$ (Fig. 2D).

### Standardization of respiration

Patients’ respirations were standardized for all ultrasound measurements. Patients were instructed first to exhale completely and then inspire through a turbohaler training whistle attached to a plastic bag with a volume of one liter (Supplemental material 1). The training whistle emits a distinct noise when reaching a fixed inspiratory pressure threshold. Hence, a fixed threshold pressure was maintained during inspiration of a one-liter volume. Ultrasound recordings were accepted if the training whistle emitted sound throughout the entire inspiration and the bag was emptied completely.

### Phrenic nerve conduction studies

Phrenic nerve conduction studies were performed using a nerve excitability testing set-up with a protocol written for this purpose (NCS-A.QRP) controlled by QtracS (Written by H. Bostock, Copyright Institute of Neurology, University College, UK). The excitability set-up consisted of a DS5 bipolar stimulator set-up, a D440 amplifier, and a HumBug noise eliminator (Digitimer Ltd., Welwyn Garden City, UK) and a Data Acquisition Device (USB-6221-BNC, National Instruments USA) [13].

Diaphragmatic compound muscle action potential (CMAP) was elicited bilaterally by percutaneous bipolar phrenic nerve stimulations of the posterior border of the clavicular head of the sternocleidomastoid muscle. The recording electrode (G1) was placed 5 cm above the xiphoid process and the reference electrode (G2) was placed 16 cm laterally from G1 along the ipsilateral costal margin [14]. Inadvertent stimulation of the brachial plexus was detected by arm movement and avoided [15]. Supramaximal stimulations were ensured by tracking the amplitude of the diaphragmatic CMAP while increasing the electrical stimulus intensity. Supramaximal stimulation was reached when the diaphragmatic CMAP did not increase despite further increases in electrical stimulation. Subsequently, supramaximal stimulations were applied over a period of approximately 15 s with 1 s stimulus intervals. The recording with the highest reproducible baseline-to-peak amplitude was selected for analyses. In addition, transmission delay was measured as the time from the onset of the stimulation artifact to the onset of the action potential. Baseline-to-peak amplitude, peak-to-to amplitude, and transmission delay were measured (Supplemental material 2).

### Spirometry

Spirometry was performed in accordance with standardized clinical guidelines [16] using a Pneumotrac Spirometer and Spirotrac 5 Software (Buckingham, England, Vitalograph). At least three measurements were performed and the highest reproduceable forced expiratory volume within the first second (FEV1) and forced vital capacity (FVC) were selected.

### 6-minute walk test

A 6-min walk test was performed in a 30-m-long corridor with continuous verbal encouragement to cover the longest distance as possible. The timer was not stopped if the patient needed to rest due to dyspnea. Instead, the patient was encouraged to continue as soon as possible with stopping the timer.

### Postoperative pulmonary complications

PPCs were obtained from the Central Denmark Region electronic patient health record system (Systematic, Aarhus, Denmark) by a physician blinded to the study protocol. PPCs comprised pneumonia, atelectasis, bronchospasm, hypoxemia, pleural effusion, pneumothorax, and use of non-invasive mechanical ventilation exceeding the first postoperative day (See Appendix 1 for definitions). PPCs were temporally confined to 30 days after surgery.

### Endpoints

The primary endpoint was change in right posterior hemidiaphragmatic excursion. We chose this excursion parameter as the primary endpoint, as it is a well-validated excursion measure and feasible and its correlation with expired lung volume is higher than seen with other excursion measures [9, 11]. Secondary ultrasound endpoints were changes in top point excursion, change in intrathoracic area and diaphragmatic thickening fraction. Phrenic nerve conduction endpoints comprised CMAP baseline-to-peak amplitude, CMAP peak-to-peak amplitude and transmission delay. Finally, the associations for all ultrasound measurements with the predefined PPCs were quantified.

### Statistical analysis

We predefined a reduction in posterior diaphragmatic excursion of 20% as clinically relevant. With a standard deviation of 36% (own data, not published) for the primary endpoint, we needed to include 36 patients (alpha = 0.05, power = 0.9, before-after paired analysis). We aimed to include 40 patients to increase power and compensate for missing data.

Within individual changes over time were analyzed with a repeated-measures multilevel mixed-effects linear regression model. To analyze the effect of surgery type, this was incorporated as an interaction term. The null hypothesis stated no change over time and a *p* value < 0.05 was considered significant as this analysis incorporates the number of repetitions. Student’s t tests were used for comparison of individual timepoints with baseline values and significance level was set to 0.05/2 = 0.025 as measurements were repeated twice. Univariable logistic regression analyses were used for quantifying the association between categorical PPCs (dependent variable) and predictors. For the total number of PPCs, a linear regression model was fitted. To analyze the effects of surgery types (VATS versus thoracotomy and esophagectomy versus lobectomy), these were incorporated in multivariable regression analyses as predictors. As the clinical endpoints spanned over many days, the largest change in predictors, from preoperative values to any of the two postoperative values, were incorporated in analyses. Associations were expressed as odds ratio (OR) or as regression coefficients with corresponding 95% confidence interval (95% CI) where appropriate. As predictive values for the different diaphragmatic excursion measures have not been tested previously on PPCs under standardized respiration, we decided to test 25%, 50%, and 75% declines for all diaphragmatic excursion measures on the association with PPCs.

Patient characteristics are presented as medians (interquartile range) for continuous variables and proportion (percentage) for categorical variables. Ultrasound and phrenic nerve conduction measurements are reported as mean with 95% confidence intervals (CI). All calculations were performed using STATA 17 software (College Station, TX, USA).

## Results

We enrolled 40 patients between August 2020 and October 2021. Baseline characteristics are shown in Table 1, and selected intraoperative data are shown in Supplemental material 3. Esophageal resection surgery was performed in 27 patients, and pulmonary lobectomy was performed in 13 patients. Thirty-four patients underwent right-sided surgery, and six patients underwent left-sided surgery. Of the 34 patients undergoing right-sided surgery, 18 patients had VATS performed. A flowchart of patient examinations is shown in Fig. 1. There was a total of 82 PPCs of which the most common were pleural effusion (*n* = 28) hypoxemia (*n* = 14), atelectasis (*n* = 10), pneumonia (*n* = 8), and prolonged use of CPAP/NIV (*n* = 11).

**Table 1:** Baseline characteristics

	*n* = 40
Age (years)	67 (61; 74)
Male (no)	29 (73%)
Height (cm)	174.5 (168.0; 178.5)
Weight (kg)	74.3 (64.5; 85.0)
BMI (kg/m^2^)	24.5 (22.2; 28.1)
Smoking status (no)	
Never smoker	19 (48%)
Ex-smoker	13 (32%)
Current smoker	8 (20%)
Comorbidity (no)	
Cardiovascular disease	8 (20%)
Diabetes mellitus	3 (8%)
COPD	5 (13%)
Asthma	3 (8%)
Restrictive lung disease	0 (0%)
Hypertension	19 (48%)
Psychiatric disease	1 (3%)
Chronic kidney disease	3 (8%)
Chronic liver disease	0 (0%)
Charlson comorbidity score	
0–3	1 (3%)
4–6	19 (48%)
7–9	13 (33%)
10–12	7 (18%)
ASA score	
I	0 (0%)
II	6 (15%)
III	34 (85%)
IIII	0 (0%)

Values are reported as median (interquartile range) or number (percentage)

*BMI *Body mass index, *COPD *chronic obstructive pulmonary disease, *ASA *American Society of Anesthesiologists

### Ultrasonographic measurements of diaphragmatic excursion and thickening fraction

**Fig. 3:**
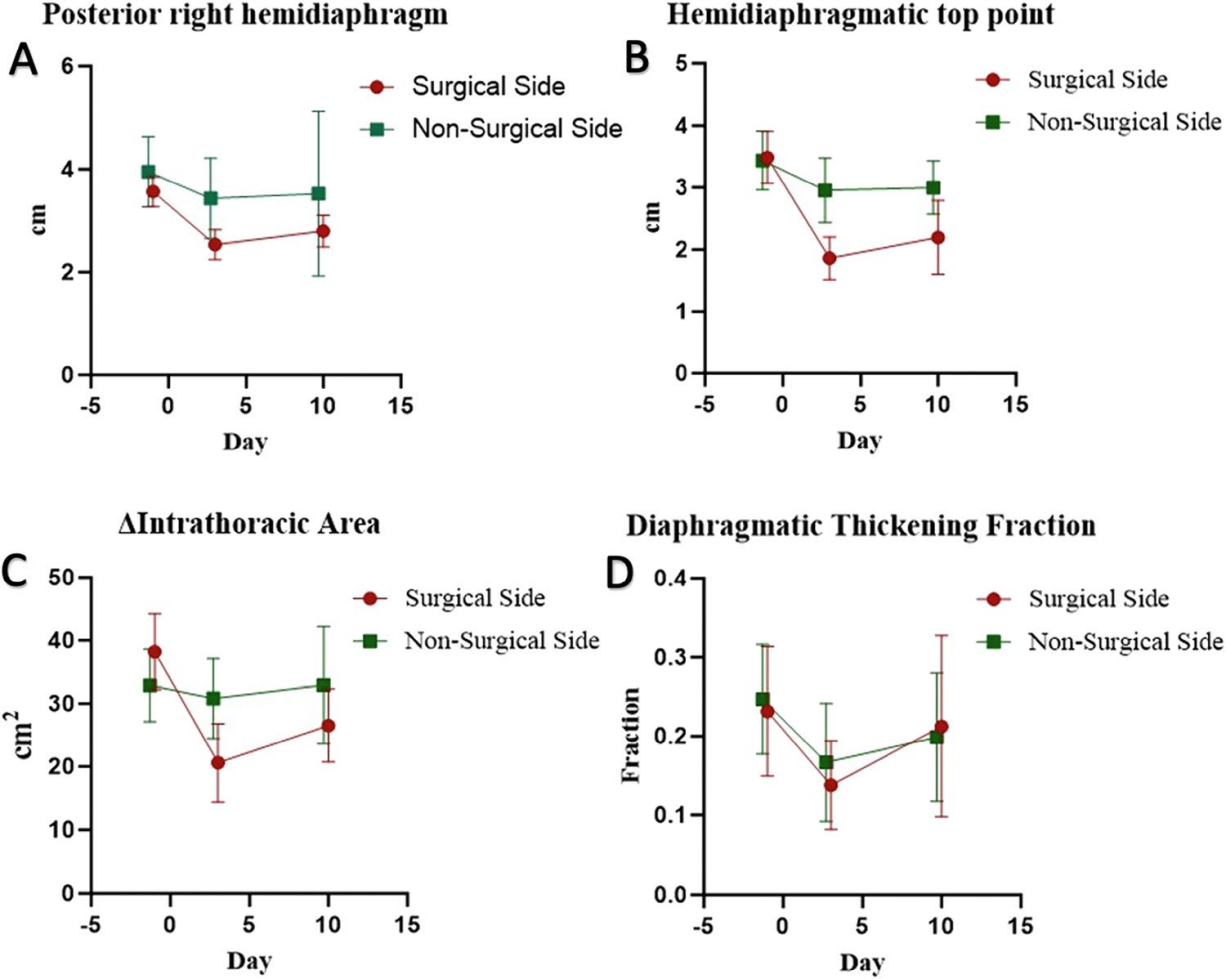
Changes in diaphragmatic ultrasound endpoints over time. **A** Posterior diaphragmatic excursion. **B** Hemidiaphragmatic top point excursion. **C** Intrathoracic area. **D** Diaphragmatic thickening fraction. Results are reported for both the surgical and non-surgical side of the thorax. *p*-values are reported as means and error brackets indicate 95% confidence intervals. The number of measurements at individual time points is reported in Fig. 1

Excursion of the right posterior hemidiaphragm changed significantly in patients operated on the right side (*p* < 0.001). There was a significant reduction in excursion from the day before surgery until 3 days after surgery (mean difference: − 1.03 cm; 95% CI [− 0.55 cm, − 1.51 cm], *p* < 0.001), which remained significantly reduced 10–14 days after surgery (mean difference: − 0.87 cm; 95% CI [− 0.42 cm, − 1.31 cm], *p* < 0.001). Conversely, the right posterior hemidiaphragmatic excursion did not change significantly in patients operated on the left side (*p* = 0.38) (Fig. 3A). There was no significant difference between the esophagectomy group and the lobectomy group for change in right posterior hemidiaphragmatic excursion over time on the surgical side (*p* = 0.18) and a similar result was found when comparing VATS to thoracotomy (*p* = 0.33).

Diaphragmatic top point excursion differed significantly over time when measured on the surgical side (*p* < 0.001). A significant reduction was observed from the preoperative assessment to 3 days after surgery (mean difference: − 1.61 cm; 95% CI [− 1.04 cm, − 2.16 cm], *p* < 0.001) and 10–14 days after surgery (mean difference: − 1.42 cm; 95% CI [− 0.48 cm, − 2.35 cm], *p* = 0.002). The top point excursion did not reach statistical significance on the non-surgical side (*p* = 0.27) (Fig. 3B). There was no significant difference between the esophagectomy group and the lobectomy group for change in diaphragmatic top point excursion over time on both the surgical side (*p* = 0.27) and non-surgical side (*p* = 0.10). Further, we did not find a significant difference between VATS and thoracotomy on the surgical side (*p* = 0.43) and the non-surgical side (*p* = 0.31) for diaphragmatic top point excursion over time.

Diaphragmatic excursion measured as change in intrathoracic area using The Area Method changed significantly on the surgical side over time (*p* < 0.001). There was a significant reduction in intrathoracic area from the day before surgery until 3 days after surgery (mean difference: − 18.9 cm^2^; 95% CI [− 12.3 cm^2^, − 25.4 cm^2^], *p* < 0.001) and the reduction was still significantly different from the baseline value 10–14 days after surgery (mean difference: − 13.3 cm^2^; 95% CI [− 5.2 cm^2^, − 21.3 cm^2^], *p* = 0.001). No statistically significant change was seen on the non-surgical side between assessments (*p* = 0.88) (Fig. 3C). There was no significant difference between the esophagectomy group and the lobectomy group for change in intrathoracic area over time on both the surgical side (*p* = 0.77) and non-surgical side (*p* = 0.19). Further, there was no significant change in intrathoracic area over time between patients undergoing VATS compared to thoracotomy on neither the surgical side (*p* = 0.33) nor the non-surgical side (*p* = 0.79).

The diaphragmatic thickening fraction did not differ significantly on neither the surgical side (*p* = 0.14) nor on the non-surgical side (*p* = 0.19) between assessments (Fig. 3D).

### Measurements of the diaphragmatic compound muscle action potential

**Fig. 4:**
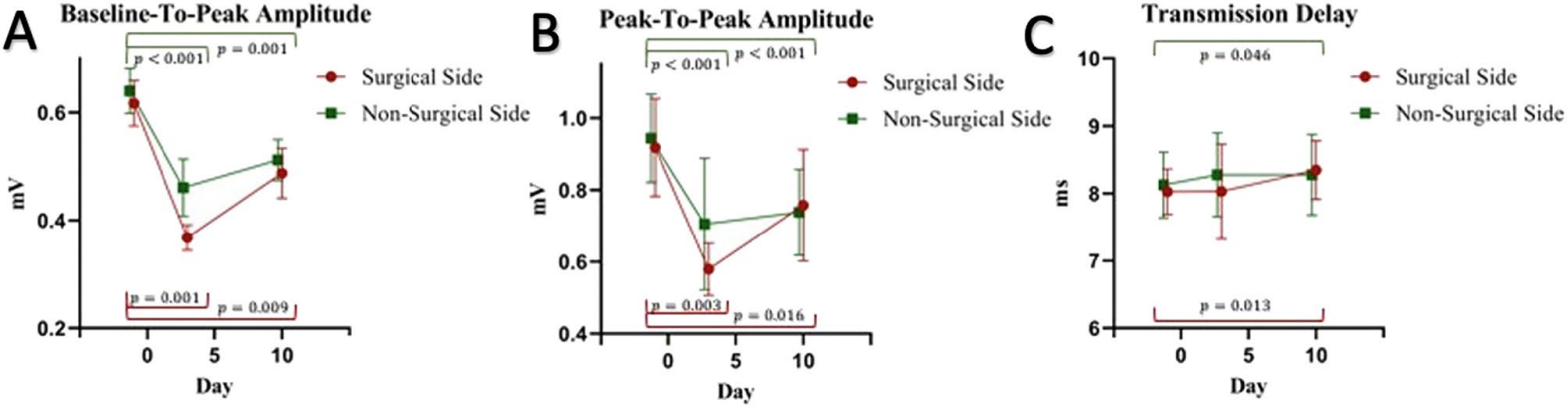
Phrenic nerve conduction measurements. **A** Diaphragmatic CMAP measured from baseline to the negative peak. **B** Diaphragmatic CMAP measured from the positive peak to the negative peak **C** Transmission delay measured as the time from the stimulation to onset of the diaphragmatic CMAP. Results are reported for both the surgical and non-surgical side of the thorax. Values are reported as mean and error brackets indicate 95% confidence interval. The number of measurements at individual time points is reported in Fig. 1

Both baseline-to-peak amplitude and peak-to-peak amplitude changed significantly on both the surgical side (both *p* values < 0.001) and the non-surgical side (both *p* values < 0.001) over time (Fig. 4).

Significant reductions were seen on the surgical side (mean difference: − 0.25 mV; 95% CI [− 0.14 mV, − 0.37 mV], *p* < 0.001) and non-surgical side (mean difference: − 0.16 mV; 95% CI [− 0.09 mV, − 0.23 mV], *p* < 0.001) 3 days after surgery for baseline-to-peak amplitude and remained significantly reduced 10–14 days after surgery on both the surgical side (mean difference: − 0.12 mV; 95% CI [− 0.03 mV, − 0.22 mV], *p* < 0.001) and the non-surgical side (mean difference: − 0.09 mV; 95% CI [− 0.04 mV, − 0.14 mV], *p* = 0.001) (Fig. 4A). There was no significant difference between the esophagectomy group and the lobectomy group for change in baseline-to-peak amplitude over time on both the surgical side (*p* = 0.82) and non-surgical side (*p* = 0.88).

Peak-to-peak amplitude was significantly reduced on both the surgical side (mean difference: − 0.36 mV; 95% CI [− 0.15 mV, − 0.57 mV], *p* < 0.001) and the non-surgical side (mean difference: − 0.24 mV; 95% CI [− 0.13 mV, − 0.35 mV], *p* < 0.001) 3 days after surgery and remained reduced 10–14 days after surgery on both the surgical side (mean difference: − 0.13 mV; 95% CI [− 0.01 mV, − 0.24 mV], *p* = 0.02) and the non-surgical side (mean difference: − 0.16 mV; 95% CI [− 0.09 mV, − 0.23 mV], *p* < 0.001) (Fig. 4B). For peak-to-peak amplitude, there was a significant difference over time between the esophagectomy group and the lobectomy group for peak-to-peak amplitude on the surgical side (difference between timepoints, *p* = 0.049) but not on the non-surgical side (difference between timepoints, *p* = 0.51).

Significant increases in transmission delay were seen on both the surgical side (*p* = 0.041) and on the non-surgical side (*p* = 0.022) between assessments, but only a small change in absolute numbers (Fig. 4C). There was no significant difference between the esophagectomy group and the lobectomy group for change in transmissions delay over time on both the surgical side (*p* = 0.90) and non-surgical side (*p* = 0.53).

### Spirometry

FEV_1_ mean decreased significantly from the baseline until 3 days after surgery (baseline mean: 2.72 L vs. 1.66 L, mean difference: 1.06 L; 95% CI [0.85 L, 1.23 L], *p* < 0.001), and remained significantly decreased 10–14 days after surgery (baseline mean: 2.72 L vs. 2.07 L, mean difference: 0.65 L; 95% CI [0.51 L, 0.91 L], *p* < 0.001). FVC also decreased significantly 3 days after surgery (baseline mean: 3.70 L vs. 2.31 L, mean difference: 1.39 L; 95% CI [1.22 L, 1.57 L], *p* < 0.001) and remained significantly decreased 10–14 days after surgery (baseline mean: 3.70 L vs. 2.89 L, mean difference: 0.91 L; 95% CI [0.70 L, 1.13 L], *p* < 0.001). There was no significant change in FEV (*p* = 0.33) or FVC (*p* = 0.36) over time between the esophagectomy group and the lobectomy group. FEV_1_/FVC ratio did not change significantly (*p* = 0.23) in the perioperative period.

### 6-min walk test distance

Walk test distance was significantly shorter 10–14 days after surgery compared to the baseline (baseline mean: 535 m vs. 492 m, mean difference: 44 m; 95% CI [21 m, 77 m], *p* < 0.001). We found no significant difference in walk test distance between the esophagectomy and lobectomy group over time (*p* = 0.82).

### Postoperative pulmonary complications

A reduction in posterior diaphragmatic excursion of > 50% at any follow-up was significantly associated with pneumonia (OR 10.7; 95% CI [1.6, 72.7], *p* = 0.018), bronchospasm (OR 9.4; 95% CI [1.2, 74.8], *p* = 0.035), hypoxemia (OR 24.7; 95% CI [2.1, 210.1], *p* = 0.010), prolonged use of CPAP/NIV (OR 11.0; 95% CI [1.6, 74.0], *p* = 0.014), and an increase in total number of postoperative pulmonary complications (coefficient: 2.69 (95% CI [1.38, 4.01], *p* < 0.001). These associations were not significantly associated with any PPCs if the cut-off for the reduction in posterior hemidiaphragmatic excursion was set at > 25%. Due to a low number of patients with a postoperative decline in posterior hemidiaphragmatic excursion of > 75% (*n* = 1), associations with PPCs for this cut-off were not considered meaningful (Table 2).

**Table 2:** Postoperative pulmonary complications if patients had $1\le$ follow-up value(s) below 25% or 50% for posterior diaphragmatic excursion

	Reduction in posterior diaphragmatic excursion of > 25% of the baseline value (*n* = 19/34)			Reduction in posterior diaphragmatic excursion of > 50% of the baseline value (*n* = 7/34)		
	Odds ratio/coefficient	95% CI	*p* value	Odds ratio/coefficient	95% CI	*p* value
Pneumonia (*n* = 8)	1.1	0.2–5.7	0.94	10.7	1.6–72.7	0.018
Atelectasis (*n* = 10)	0.5	0.1–2.5	0.42	5.9	1.0–34.9	0.052
Bronchospasm (*n* = 6)	3.7	0.4–37.6	0.29	9.4	1.2–74.8	0.035
Hypoxemia (*n* = 14)	2.0	0.5–8.6	0.35	24.7	2.1–210.1	0.010
Pleural effusion (*n* = 28)	2.7	0.5–13.7	0.20	1 (all patients in this group had pleural effusion)		–
Pneumothorax (prolonged drainage time) (*n* = 5)	0.2	0.02–2.4	0.22	1.3	0.1–15.2	0.82
Pneumothorax (renewed drainage) (*n* = 0)	1		–	1		–
CPAP/NIV after 1. Postoperative day (*n* = 11)	1.3	0.3–5.7	0.76	11.0	1.6–74.0	0.014
Total number of PPCs (*n* = 82)	0.26	− 1.06 to 1.59	0.69	2.69	1.38–4.01	< 0.001

Bold texture indicates significant P values

Values are reported as odds ratio or coefficients for logistic regression (categorical endpoints)- and linear regression (continuous endpoints) analyses, respectively, with 95% confidence interval

*CPAP *Continuous positive airway pressure, *NIV *non-invasive ventilation, *PPC *postoperative pulmonary complication

Incorporating the effects of VATS versus thoracotomy and esophagus surgery versus lobectomy in multivariable regression analyses did not have any significant influence on the associations between diaphragmatic excursion and postoperative pulmonary complications.

For the associations between diaphragmatic top point excursion and change in intrathoracic area with PPCs, see Supplemental materials 4 & 5.

## Discussion

This study unequivocally showed that diaphragmatic excursion was affected by thoracic surgery on the surgical side of the thorax for all ultrasonographic excursion measures. This is the first study to show reduced diaphragmatic function following thoracic surgery measured by both ultrasound evaluation of diaphragmatic excursion and phrenic nerve conduction indices. Furthermore, our study is the first to include multiple ultrasound measures of diaphragmatic function. We found posterior diaphragmatic excursion, measured with M-mode, to be significantly associated with PPCs when measured on the surgical side of the thorax.

The ultrasound examinations of diaphragmatic function showed reductions in most parameters. The M-mode examination performed in the midclavicular line found reduced excursion of the posterior right hemidiaphragm. The B-mode approach and The Area Method performed on both hemidiaphragms found reduced excursion on the ipsilateral side of surgery but not on the contralateral side, which indicates damage caused by the surgical trauma on the relevant hemidiaphragm. All ultrasound excursion measures increased from the third postoperative day until 10–14 days after surgery but were still significantly reduced compared to the baseline value. This indicates that diaphragmatic dysfunction is present for more than 10–14 days after thoracic surgery on the surgical side.

The most significant changes in diaphragmatic excursion were the change in intrathoracic area and diaphragmatic top point excursion. The change in intrathoracic area was performed using the area method, which is the only measure that quantifies diaphragmatic excursion in two dimensions, thus being a more comprehensive method as it incorporates both cranio-caudal and anterior–posterior movements. The diaphragmatic top point excursion quantifies cranio-caudal movement. Conversely, the M-mode approach is used to trace the motion of a chosen point on the posterior part of the diaphragm towards an anteriorly placed transducer and predominantly follows anterior–posterior movement. Therefore, our results suggest that both cranio-caudal and anterior–posterior motions are reduced after thoracic surgery, although the anterior–posterior motion was more convincingly correlated with PPCs. The positive association between posterior diaphragmatic excursion and PPCs is in accordance with previous findings after open pulmonary lobectomy compared to VATS [5].

We also measured the diaphragmatic thickening fraction, which has been used in the previous studies to predict successful weaning from mechanical ventilation when performed at deep breathing [17]. We did not find a significant change in DTf between assessments on either side of the thorax, which may be explained by the respiratory standardization, as the patients in our study did not inspire maximally during the ultrasound examination, thus limiting muscular contraction.

We standardized both the volume and the pressure of the inspiration during measurements in favor of quiet breathing or a deep sniff [5, 9] to minimize variation caused by patient-understanding, postoperative pain and fatiguing medication. Hence, the changes in diaphragmatic function were not caused by alterations in inspired volume or inspiratory pressure but were caused by changes in nervous- or muscular function.

We found a highly significant bilateral reduction in diaphragmatic CMAP amplitude 3 days after surgery. These findings may be interpreted as a significant postoperative conduction block of functional axons bilaterally as the CMAP amplitude idealizes the summation of simultaneous action potentials within the targeted area [18]. The change was most prominent on the surgical side of the thorax, suggesting that the postoperative loss may in part be conditional upon surgical manipulation. However, phrenic nerve measures from the non-surgical side were also significantly affected and may be a result of a generalized surgical inflammatory stress response or systemic affection rather than direct surgical nerve damage. This seems explicable, as reversible conduction block is a well-recognized phenomenon in acute inflammatory neuropathies [19]. However, 2/3 of the patient cohort underwent esophageal resection, which is performed in close relation to both phrenic nerves. In these patients, surgical manipulation may affect the phrenic nerves bilaterally. There was, however, no significant effect of surgery type on phrenic nerve conduction over time which favors a generalized systemic affection rather than surgical manipulation to the non-surgical side of phrenic nerve conduction. Thoracic epidural anesthesia has further been suggested to affect phrenic nerve conduction, possibly by inhibition of reflex pathways at the spinal root level [20].

The changes in phrenic nerve conduction seen on the non-surgical side were not mirrored in ultrasound changes in diaphragmatic excursion. There may be several explanations for this. First, it may be that there was no causal relationship between reduced phrenic nerve conduction and changes in diaphragmatic excursion. Second, it may have been due to the fixed inspiratory volume and inspiratory pressure threshold utilized to minimize random errors. Consequently, diaphragmatic excursion was not measured at maximal excursion, whereas phrenic nerve conduction measures were performed under supramaximal stimulation. Therefore, the insignificant reduction in diaphragmatic excursion on the non-surgical side may have yielded different results if ultrasound measurements were performed with higher inspiratory effort.

Subanalyses for the effects of surgery type, i.e., VATS versus thoracotomy and esophagectomy versus lobectomy, showed that some PPCs were more common in patients undergoing thoracotomy (data not shown), which is in line with a previous study [5]. However, when incorporating surgery type into multivariable analyses, surgery type did not alter the results of the association between reduced diaphragmatic excursion and PPCs. Hence, from a clinical endpoint, one does not have to take into account the surgery type if low diaphragmatic excursion is found and a higher risk of PPCs can be expected. In line with this, we did not find a significant effect of surgery type on 6-min walk distance or spirometry parameters.

Several limitations to our study must be acknowledged. First, the primary endpoint, excursion of the posterior diaphragm, was not performed on the left side due to obstructing air–fluid content in the stomach [15]. However, measures of top point excursion and difference in intrathoracic area showed similar results and were performed on both sides, which lowers the redundancy of this limitation. Second, we found transcutaneous phrenic nerve stimulation unfeasible in some patients, especially those with short necks or a BMI > 30 kg/m^2^. In addition, a substantial part of the patients refused to participate in the phrenic nerve conduction studies, especially in the early days after surgery, as motoric nerve stimulations are moderately uncomfortable. These postoperative dropouts raise concerns about an underestimation of the degree of phrenic nerve paresis compared to the general surgical population, as patients who suffered from postoperative complications were reluctant to undergo phrenic nerve stimulations. Thus, it was predominantly patients with uncomplicated admissions who participated in the phrenic nerve conduction studies. Finally, there were missing data on diaphragmatic excursion endpoints due to patient discharges and patients' preferences. This may have increased the risk of type II error.

The perspectives from the study merit more focus on diaphragmatic function in patients undergoing thoracic surgery as our results prove a relationship between diaphragmatic dysfunction and PPCs. Ultrasound assessment of diaphragmatic function is easily and quickly performed. Integration of diaphragmatic ultrasound could become an integral part of predicting PPCs after thoracic surgery, but more studies with larger populations are needed to specify the precise role in the perioperative course.

## Conclusion

Diaphragmatic excursion was significantly reduced 3 days after thoracic surgery and, to a lesser extent, 10 days after surgery, across all ultrasonographic excursion measures on the surgical side of the thorax. A reduction in excursion of the posterior hemidiaphragm on the surgical side of more than 50% was strongly associated with PPCs. All indices of phrenic nerve conduction were highly significantly affected by surgery. Therefore, our findings show that diaphragmatic dysfunction is accompanied by phrenic nerve paresis after thoracic surgical procedures, which may be a key factor in the pathogenesis of PPCs.

### Supplementary Information

Below is the link to the electronic supplementary material.

Supplementary file1 (DOCX 13 kb)

Supplementary file2 (DOCX 574 kb)

## Data Availability

Data will be shared in deidentified form upon reasonable request.

## References

[1] Pedoto A, Perrino AC (2019). Delayed recovery following thoracic surgery: persistent issues and potential interventions. Curr Opin Anaesthesiol.

[2] Dimick JB, Chen SL, Taheri PA, Henderson WG, Khuri SF, Campbell DA (2004). Hospital costs associated with surgical complications: a report from the private-sector national surgical quality improvement program. J Am Coll Surg.

[3] Fernandez-Bustamante A, Frendl G, Sprung J, Kor DJ, Subramaniam B, Martinez Ruiz R, Lee JW, Henderson WG, Moss A, Mehdiratta N, Colwell MM, Bartels K, Kolodzie K, Giquel J, Vidal Melo MF (2017). Postoperative pulmonary complications, early mortality, and hospital stay following noncardiothoracic surgery: a multicenter study by the perioperative research network investigators. JAMA Surg.

[4] Khuri SF, Henderson WG, DePalma RG, Mosca C, Healey NA, Kumbhani DJ (2005). Determinants of long-term survival after major surgery and the adverse effect of postoperative complications. Ann Surg.

[5] Spadaro S, Grasso S, Dres M, Fogagnolo A, Dalla Corte F, Tamburini N, Maniscalco P, Cavallesco G, Alvisi V, Stripoli T, De Camillis E, Ragazzi R, Volta CA (2019). Point of care ultrasound to identify diaphragmatic dysfunction after thoracic surgery. Anesthesiology.

[6] Kocher GJ, Mauss K, Carboni GL, Hoksch B, Kuster R, Ott SR, Schmid RA (2013). Effect of phrenic nerve palsy on early postoperative lung function after pneumonectomy: a prospective study. Ann Thorac Surg.

[7] Efthimiou J, Butler J, Woodham C, Benson MK, Westaby S (1991). Diaphragm paralysis following cardiac surgery: role of phrenic nerve cold injury. Ann Thorac Surg.

[8] Hansen HJ, Petersen RH (2012). Video-assisted thoracoscopic lobectomy using a standardized three-port anterior approach - the Copenhagen experience. Ann Cardiothorac Surg.

[9] Boussuges A, Gole Y, Blanc P (2009). Diaphragmatic motion studied by m-mode ultrasonography: methods, reproducibility, and normal values. Chest.

[10] Gethin-Jones TL, Noble VE, Morse CR (2010). Quantification of diaphragm function using ultrasound: evaluation of a novel technique. Ultrasound Med Biol.

[11] Skaarup SH, Løkke A, Laursen CB (2018). The Area method: a new method for ultrasound assessment of diaphragmatic movement. Crit Ultrasound J.

[12] Carrillo-Esper R, Pérez-Calatayud ÁA, Arch-Tirado E, Díaz-Carrillo MA, Garrido-Aguirre E, Tapia-Velazco R, Peña-Pérez CA, Espinoza-de Los Monteros I, Meza-Márquez JM, Flores-Rivera OI, Zepeda-Mendoza AD, Torre-León TDL (2016). Standardization of sonographic diaphragm thickness evaluations in healthy volunteers. Respir Care.

[13] Bostock H, Cikurel K, Burke D (1998). Threshold tracking techniques in the study of human peripheral nerve. Muscle Nerve.

[14] Vincent M, Court-Fortune I, Costes F, Antoine JC, Camdessanché JP (2019). Phrenic nerve conduction in healthy subjects. Muscle Nerve.

[15] Bolton CF (1993). AAEM minimonograph #40: clinical neurophysiology of the respiratory system. Muscle Nerve.

[16] Graham BL, Steenbruggen I, Miller MR, Barjaktarevic IZ, Cooper BG, Hall GL, Hallstrand TS, Kaminsky DA, McCarthy K, McCormack MC, Oropez CE, Rosenfeld M, Stanojevic S, Swanney MP, Thompson BR (2019). Standardization of spirometry 2019. Update an official american thoracic society and European respiratory society technical statement. Am J Respir Crit Care Med.

[17] Samanta S, Singh RK, Baronia AK, Poddar B, Azim A, Gurjar M (2017). Diaphragm thickening fraction to predict weaning-a prospective exploratory study. J Intensive Care.

[18] Tavee J (2019). Nerve conduction studies: basic concepts. Handb Clin Neurol.

[19] Kokubun N, Nishibayashi M, Uncini A, Odaka M, Hirata K, Yuki N (2010). Conduction block in acute motor axonal neuropathy. Brain.

[20] Chae WS, Choi S, Sugiyama D, Richerson GB, Brennan TJ, Kang S (2018). Effect of thoracic epidural anesthesia in a rat model of phrenic motor inhibition after upper abdominal surgery. Anesthesiology.

