# Supplementary material for: Diaphragmatic dysfunction is associated with postoperative pulmonary complications and phrenic nerve paresis in patients undergoing thoracic surgery
Source: J Anesth. 2024 Mar 28;38(3):386–97. doi: 10.1007/s00540-024-03325-5 (PMC11096220; doi:10.1007/s00540-024-03325-5)
Supplement: Supplementary file 1 — Supplementary file1 (DOCX 13 kb) [file 540_2024_3325_MOESM1_ESM.docx]

# Definition of postoperative pulmonary complications

**Pneumonia:** In case patient receives antibiotics and meets at least one of the following criteria: new or changed sputum, new or changed lung opacities on chest X-ray when clinically indicated, tympanic temperature > 38·3°C, WBC count > 12 x10^9/L.

**Atelectasis:** Suggested by lung opacification with shift of the mediastinum, hilum, or hemidiaphragm towards the affected area, and compensatory overinflation in the adjacent non-atelectatic lung (excluding xray on the day of surgery).

**Bronchospasm:** Newly detected expiratory wheezing treated with bronchodilators.

**Hypoxemia:** Pao2 less than 8kPa or oxygen saturation measured by pulse oximetry less than 90% in room air but responding to supplemental oxygen (excluding hypoventilation).

**Pleural Effusion:** Chest X-ray demonstrating blunting of the costophrenic angle, loss of the sharp silhouette of the ipsilateral hemidiaphragm in upright position, evidence of displacement of adjacent anatomical structures, or (in supine position) a hazy opacity in one hemi-thorax with preserved vascular shadows - OR Ultrasonographic confirmation of pleural effusion > 1 cm.

**Pneumothorax:** Drain in situ exceeding 5 days for pulmonary lobectomy or 8 days for esophageal resection (If no anastomotic leakage on day 8).

**Pneumothorax:** Requiring renewed drainage.

**CPAP or NIV required after first postoperative day:** In addition to the standard treatment.
