# Supplementary material for: Diaphragmatic dysfunction is associated with postoperative pulmonary complications and phrenic nerve paresis in patients undergoing thoracic surgery
Source: J Anesth. 2024 Mar 28;38(3):386–97. doi: 10.1007/s00540-024-03325-5 (PMC11096220; doi:10.1007/s00540-024-03325-5)
Supplement: Supplementary file 2 — Supplementary file2 (DOCX 574 kb) [file 540_2024_3325_MOESM2_ESM.docx]

# Supplemental material

Supplemental material 1


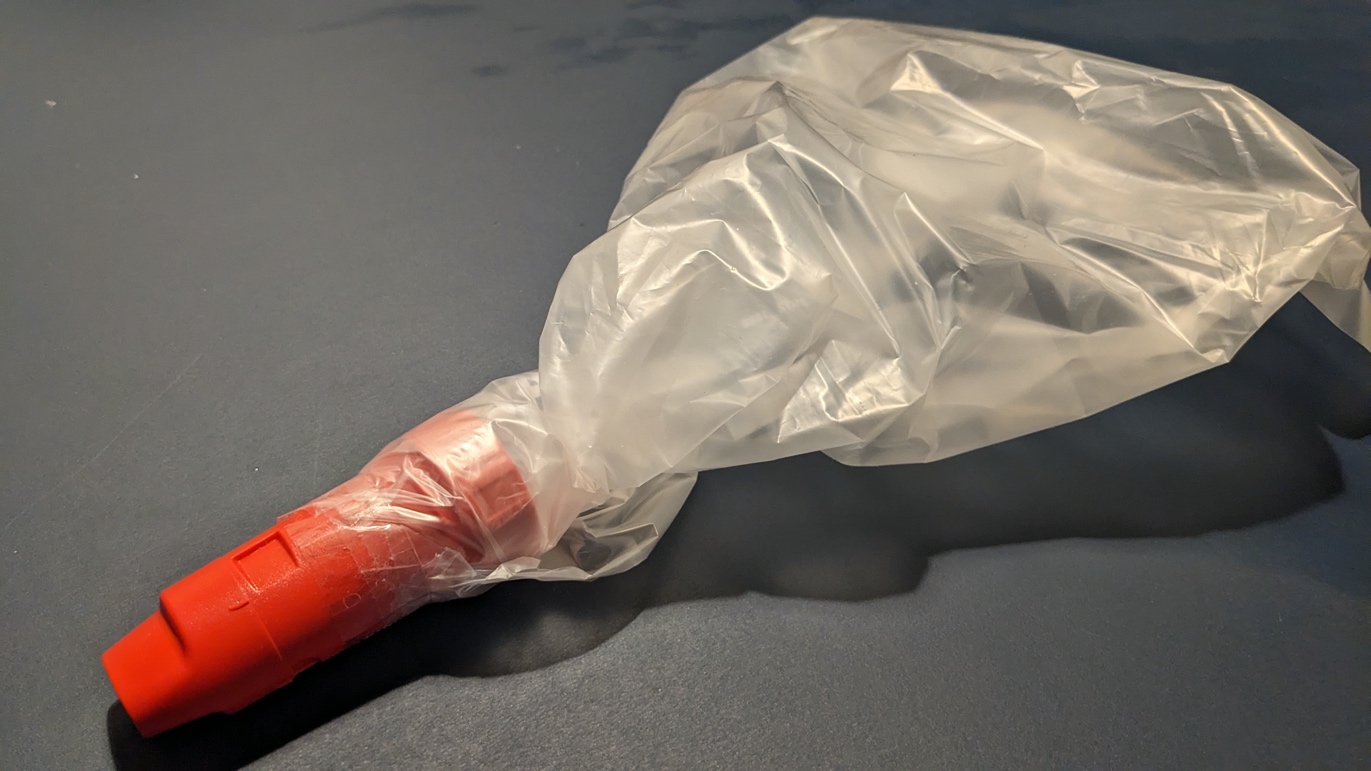


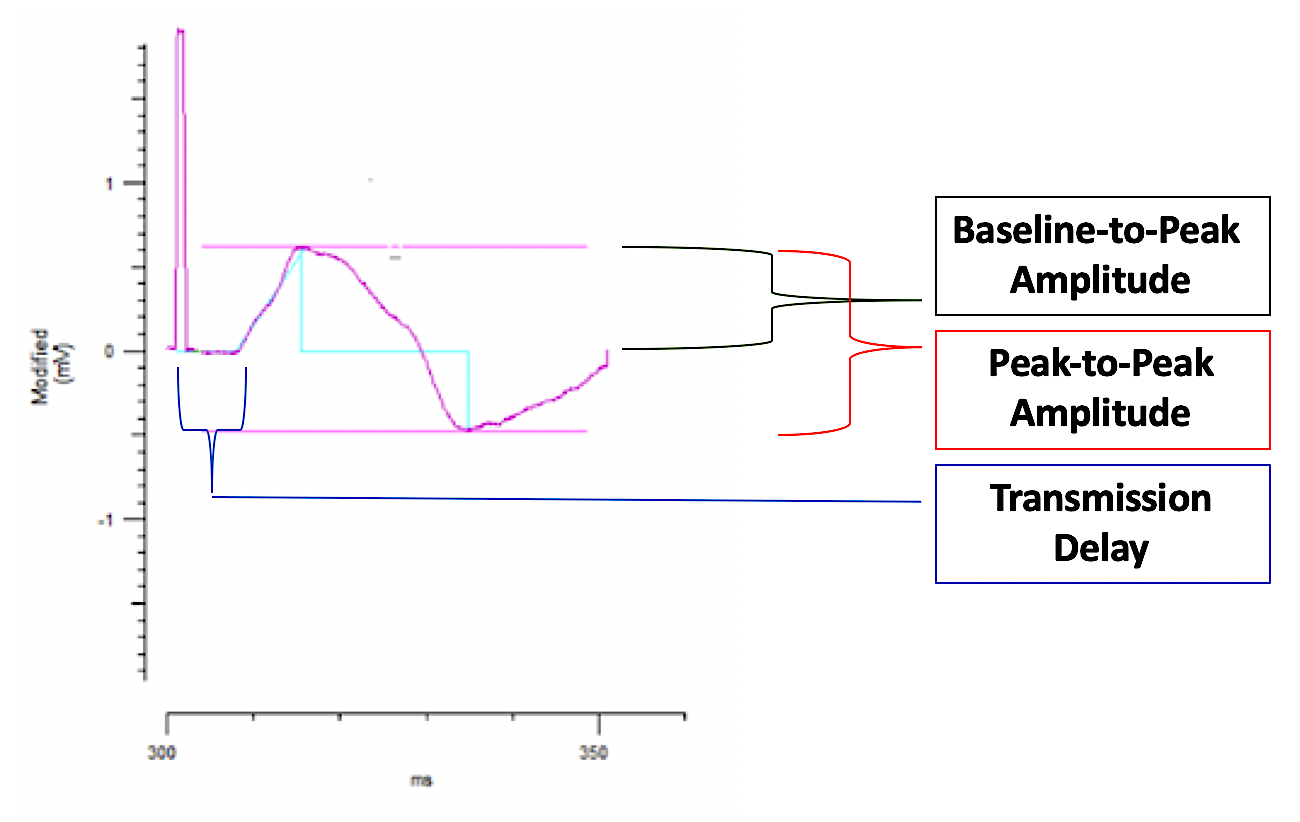
Supplemental material 2

Supplemental material 3

|  | **Pulmonary Lobectomy (n= 13)** | **Esophageal Resection (n = 27)** |
| --- | --- | --- |
| **Duration of anesthesia (minutes)** | 203 (175; 232) | 378 (355; 400) |
| **Duration of surgery (minutes)** | 131 (102; 160) | 291 (265; 317) |
| **Duration of one-lung ventilation (minutes)** | 114 (86; 143) | 116 (101; 130) |
| **Max FiO_2_ applied (O_2_ fraction)** | 54 (48; 61) | 64 (58; 71) |
| **Max PEEP applied (cm H_2_O)** | 5 (4; 6) | 5 (4; 6) |
| **Open thoracic surgery (no)** | 4 (31%) | 13 (48%) |
| **Surgery converted to open (from VATS)** | 0 (0%) | 3 (11%) |
| **Total PPCs (no)** | 16 | 66 |
| **Pneumonia (no)** | 2 (15%) | 6 (22%) |
| **Atelectasis (no)** | 1 (8%) | 9 (33%) |
| **Bronchospasm (no)** | 1 (8%) | 5 (19%) |
| **Hypoxemia (no)** | 4 (31%) | 10 (37%) |
| **Pleural Effusion (no)** | 4 (31%) | 24 (89%) |
| **Pneumothorax (prolonged drainage time) (no)** | 3 (23%) | 2 (7%) |
| **CPAP/NIV after 1. Postoperative day (no)** | 1 (8%) | 10 (37%) |

Supplemental material 4

|  | **Reduction in top point excursion of >25% of the baseline value (n = 28/36)** | | | **Reduction in top point excursion of >50% of the baseline value (n = 10/36)** | | | **Reduction in top point excursion of >75% of the baseline value (n = 10/36)** | | |
| --- | --- | --- | --- | --- | --- | --- | --- | --- | --- |
|  | **Odds Ratio** | **95% CI** | **P-value** | **Odds Ratio** | **95% CI** | **P-value** | **Odds Ratio** | **95% CI** | **P-value** |
| **Pneumonia (n = 8)** | 0.7 | 0.1-4.2 | 0.65 | 1.2 | 0.2-6.6 | 0.80 | 2.4 | 0.4-13.2 | 0.33 |
| **Atelectasis (n = 10)** | 0.5 | 0.1-2.5 | 0.36 | 1.2 | 0.3-5.3 | 0.85 | 2.8 | 0.6-13.8 | 0.21 |
| **Bronchospasm (n = 6)** | 1 | - | - | 3.0 | 0.3-32.0 | 0.36 | 0.9 | 0.8-9.3 | 0.90 |
| **Hypoxemia (n = 14)** | 1.4 | 0.2-8.5 | 0.70 | 1.4 | 0.2-8.5 | 0.70 | 6.3 | 1.3-31.1 | **0.024** |
| **Pleural Effusion (n = 28)** | 0.3 | 0.0-2.4 | 0.23 | 0.5 | 0.1-2.3 | 0.39 | 1.0 | 0.2-5.1 | 0.96 |
| **Pneumothorax (prolonged drainage time) (n = 5)** | 1 | - | - | 4.2 | 0.4-42.6 | 0.82 | 5.1 | 0.7-37.2 | 0.10 |
| **Pneumothorax (Renewed drainage) (n = 0)** | 1 | - | - | 1 | - | - | 1 | - | - |
| **CPAP/NIV after 1. Postoperative day (n = 11)** | 0.5 | 0.1-2.5 | 0.36 | 0.6 | 0.1-2.9 | 0.57 | 0.7 | 0.1-4.0 | 0.67 |
| **Total number of PPCs (n = 82)** | 0.9 | 0.6-1.4 | 0.59 | 1.1 | 0.8-1.6 | 0.58 | 1.3 | 0.9-1.9 | 0.19 |

Supplemental material 5

|  | **Reduction in change in intrathoracic area of >25% of the baseline value (n = 29/34)** | | | **Reduction in change in intrathoracic area of >50% of the baseline value (n = 18/36)** | | | **Reduction in change in intrathoracic area of >75% of the baseline value (n = 8/36)** | | |
| --- | --- | --- | --- | --- | --- | --- | --- | --- | --- |
|  | **Odds Ratio** | **95% CI** | **P-value** | **Odds Ratio** | **95% CI** | **P-value** | **Odds Ratio** | **95% CI** | **P-value** |
| **Pneumonia (n = 8)** | 1 | - | - | 2.7 | 0.4-16.1 | 0.28 | 6.4 | 1.1-38.0 | **0.041** |
| **Atelectasis (n = 10)** | 1.0 | 0.2-6.1 | 1 | 1.2 | 0.3-5.3 | 0.85 | 1.8 | 0.3-9.2 | 0.51 |
| **Bronchospasm (n = 6)** | 1 | - | - | 3.0 | 0.3-32.0 | 0.36 | 13.0 | 1.1-147.8 | **0.039** |
| **Hypoxemia (n = 14)** | 1.4 | 0.2-8.5 | 0.70 | 3.4 | 0.7-15.9 | 0.12 | 8.8 | 1.6-47.8 | **0.012** |
| **Pleural Effusion (n = 28)** | 0.7 | 0.1-4.2 | 0.70 | 1.5 | 0.4-6.4 | 0.56 | 4.7 | 0.5-43.4 | 0.17 |
| **Pneumothorax (prolonged drainage time) (n = 5)** | 1.2 | 0.1-12.3 | 0.90 | 1.4 | 0.2-9.6 | 0.73 | 0.7 | 0.1-7.4 | 0.78 |
| **Pneumothorax (Renewed drainage) (n = 0)** | 1 | - | - | 1 | - | - | 1 | - | - |
| **CPAP/NIV after 1. Postoperative day (n = 11)** | 1.0 | 0.2-6.1 | 1 | 1.2 | 0.3-5.3 | 0.85 | 3.5 | 0.7-18.0 | 0.13 |
| **Total number of PPCs (n = 82)** | 1.1 | 0.7-1.8 | 0.58 | 1.2 | 0.8-1.8 | 0.28 | 1.6 | 1.1-2.6 | **0.026** |

# Supplemental material legends

**Supplemental material 1:** Constant flow and volume limited inhalation device (CFVLID) with a volume of one liter for standardization of respiration. Patients were instructed first to exhale completely and then inspire through the turbohaler until the bag was empty.

**Supplemental material 2: The diaphragmatic compound muscle action potential for quantification of phrenic nerve function:** The amplitude was measured from baseline to the negative peak and from the negative peak to the positive peak, respectively. Transmission delay was measured from the stimulation artefact to the onset of the negative amplitude peak. ms = milliseconds, mV = millivolt.

**Supplemental material 3: Perioperative Data.** Values are reported as median (interquartile range) or number (percent). FiO_2_ = Fraction of inspired oxygen. PEEP = Positive end expiratory pressure. VATS = Video-assisted thoracoscopic surgery.

**Supplemental material 4: Postoperative pulmonary complications if patients had** $\boldsymbol{1\leq}$ **follow-up value(s) below 25%, 50% or 75% of the baseline value for top point excursion on the surgical side.** Values are reported as odds ratio with 95% confidence interval. CPAP = Continuous positive airway pressure. NIV = Non-invasive ventilation. PPC = Postoperative pulmonary complication.

**Supplemental material 5: Postoperative pulmonary complications if patients had** $\boldsymbol{1\leq}$ **follow-up value(s) below 25%, 50% or 75% of the baseline value for intrathoracic area on the surgical side.** Values are reported as odds ratio with 95% confidence interval. CPAP = Continuous positive airway pressure. NIV = Non-invasive ventilation. PPC = Postoperative pulmonary complication.
